# Association of high-density lipoprotein with development of metabolic syndrome components: a five-year follow-up in adults

**DOI:** 10.1186/s12889-015-1747-9

**Published:** 2015-04-22

**Authors:** Xiangtong Liu, Lixin Tao, Kai Cao, Zhaoping Wang, Dongning Chen, Jin Guo, Huiping Zhu, Xinghua Yang, Youxin Wang, Jingjing Wang, Chao Wang, Long Liu, Xiuhua Guo

**Affiliations:** School of Public Health, Capital Medical University, No. 10 Xitoutiao, You’anmen Wai, Fengtai District, Beijing, 100069 China; Beijing Municipal Key Laboratory of Clinical Epidemiology, Beijing, 100069 China; Physical Examination Department, Beijing Tongren Hospital Affiliated to Capital Medical University, No.1 Dongjiao Minxiang, Dongcheng District, Beijing, 100730 China

**Keywords:** Metabolic syndrome, High-density lipoprotein, Follow-up study, Adults

## Abstract

**Background:**

High-density lipoprotein (HDL) is associated with the incidence of metabolic syndrome (MetS). It is unclear whether subjects with different HDL levels develop different components of MetS over time. Our study aimed to determine what MetS components tend to emerge and change relative to different levels of HDL.

**Methods:**

During the period 2007 to 2012, 4,905 adults in Tongren and Xiaotangshan Hospitals in Beijing were included with no MetS, self-reported type 2 diabetes, or cardiovascular disease at baseline. An association rule was used to determine the changes of MetS components over time.

**Results:**

The incidence of MetS at follow-up was 3.40% for men and 1.50% for women in the high-normal HDL group; 6.65% and 4.55%, respectively, in the normal HDL group; and 11.05% and 6.45%, respectively, in the low HDL group. The most common transition was from healthy to healthy in normal-high or normal HDL groups (47.2% to 63.8%), whereas 11.7% to 39.9% of subjects with low HDL returned to healthy status or stayed unchanged in the low HDL group. The most common new-onset components were elevated blood pressure (9.2 to 10.0%), elevated high-density lipoprotein (5.5 to 11.0%), and raised fasting glucose (5.4 to 5.5%) in the groups with normal-high or normal HDL.

**Conclusions:**

The incidence of MetS increased in parallel with the decrease in HDL. Adults with a low HDL level were more susceptible to developing MetS over time. Low HDL seemed to be a pre-existing phase of MetS and may be a crucial status for MetS prevention.

## Background

Metabolic syndrome (MetS) is a complicated medical condition that includes five components: high plasma glucose, high triglycerides, low high-density lipoprotein cholesterol, high blood pressure, and abdominal obesity. MetS is associated with an increased risk of the development of cardiovascular disorders worldwide. Patients with MetS have a two-fold greater relative risk for cardiovascular disease (CVD), a five-fold greater risk for type 2 diabetes mellitus (DM), a 2.2-fold greater risk for stroke, and a 2.6-fold greater risk for chronic kidney disease [1-3]. A meta-analysis of 172,573 individuals demonstrated a significantly higher risk of cardiovascular events associated with MetS [4]. Lipid abnormalities, including low high-density lipoprotein (HDL), in MetS may contribute substantially to these high cardiovascular risks [5].

It is not yet clear whether or not MetS has a single cause, although it is known that insulin resistance is a major underlying cause of MetS [6-8]. HDL has been shown to be a key component in the prediction of CVD risk and may trigger the pathogenesis of MetS [9]. A cross-sectional study indicated that the HDL sub-class ratio is strongly associated with HDL, insulin resistance, as well as other MetS components in Japanese individuals [10]. However, there have been few cohort studies investigating which MetS components tend to cluster dominantly, and how the status of each component changes over time [11-13]. These factors may hamper the adequacy of management among individuals with MetS. Our previous study revealed that people with optimal and normal blood pressure were less susceptible to developing MetS over a five year period, whereas abnormal blood pressure seemed to be a pre-existing phase of MetS [14].

Multiple statistical methods, such as logistical regression, Cox proportional hazards regression model, a net-work approach, latent transition, and factor analysis were applied in the previous research [8,9,11-13]. However, these methods can not deal with independent variables that are correlated with each other. Thus association rule mining (ARM), which is a popular data mining method designed to identify groups of variables that are strongly correlated with each other, or with respect to a specific target variable, was used in the present study [15].

The purposes of this study were to investigate: 1) whether or not there is a correlation between the level of HDL and the incidence of MetS; 2) which components tend to develop at each level of HDL; and 3) how these components change with time.

## Methods

### Survey methodology

Subjects’ health records from routine physical check-ups have been computerised since 2007 at Beijing Tongren and Xiaotangshan Hospitals. We selected the records of adult visitors who had a physical check-up, using entries in 2007/2008 as the baseline, and 2011/2012 as the end point. In total, 6,888 people who had check-up records at both baseline and the follow-up were enrolled. Firstly, 438 people with any medical history of stroke, cerebral infarction, myocardial infarction, or coronary heart disease, or in use of any hypoglycaemic, anti-dislipidemic, or anti-hypertensive treatments were excluded. Secondly, 13 people aged less than 20 years and 991 people aged over 65 years were excluded. Lastly, 538 subjects who had MetS at baseline were ruled out. The final cohort was composed of 4,905 subjects.

### Measurements and laboratory tests

The participants underwent routine physical examinations including measurements of weight, height, blood pressure (BP), and analyses of blood biochemistry parameters. Height and body weight were measured in the upright position to the nearest of 0.5 cm and 0.1 kg, respectively. Electronic sphygmomanometers were used by a trained nurse to measure the BP of each subject in the sitting position after a 10-minute rest period. During the 30 min preceding the measurements, the subjects were required to refrain from smoking or consuming caffeine [16]. The readings of systolic blood pressure (SBP) and diastolic blood pressure (DBP) were recorded, when the second measurement was in an extent of 5 mmHg. Three systolic and diastolic blood pressures were recorded, with an interval of at least 1 minute between readings, and the average of the last two measurements was used for data analysis [16].

Blood samples were collected from an antecubital vein in the morning after an overnight fasting period and placed in tubes containing EDTA. The samples were analysed immediately after pre-treatment or stored at -80°C in the ISO 15189 accredited medical laboratories of the hospital for further analysis. Serum HDL concentration was measured photometrically (Hitachi 704; Roche, Mannheim, Germany), and TG and FPG concentrations were determined enzymatically (Hitachi 717; Roche Diagnostics). All analyses were performed in accordance with the manufacturer’s recommendations. The intra- and inter-assay coefficients of variation for all laboratory tests were under 5%.

### Diagnosis of metabolic syndrome

Diagnostic criteria for the assessment of MetS components were determined according to the Joint Interim Statement Criteria [17]. Body mass index (BMI) was regarded as a substitute for the component of obesity, which was strongly correlated with waist circumference (WC) in patients with MetS [18], as WC was not obtained. The parameters used were as follows:

Overweight: 25 ≤ BMI < 30 kg/m^2^; obese: BMI ≥ 30 kg/m^2^ [19];

- Raised TG level (drug treatment for raised triglyceride level is an alternative indicator) ≥ 150 mg/dL (1.7 mmol/L) [17];
- Reduced HDL level (drug treatment for reduced HDL level is an alternative indicator): < 40 mg/dL (1.0 mmol/L) in men; < 50 mg/dL (1.3 mmol/L) in women [17];
- Raised BP (anti-hypertensive drug treatment in a patient with a history of hypertension is an alternative indicator ): systolic pressure ≥ 130 and/or diastolic ≥85 mm Hg [17];
- Raised FPG level (drug treatment with raised glucose is an alternative indicator) ≥ 100 mg/dL (5.6 mmol/L) [17];
- Participants fulfilling at least three out of these five components were diagnosed as having MetS.

### Association rule mining (ARM)

ARM, also known as market basket analysis (MBA), is a popular data mining method designed to identify groups of variables with respect to a specific target variable [15]. The strength of this method is that ARM measures the support and confidence, as explained below. The support rate of transitions is defined as the percentage of initial status to another status in all possible transitions, and the confidence rate represents the number of cases transitioned within a certain status. In this study, support rate and confidence rate were used to evaluate which were the relatively frequent transition patterns and which status increased most, respectively. More details on the *a priori* algorithm underpinning ARM are available elsewhere [20-22].

### Layered approach for high-density lipoprotein levels at baseline

HDL at baseline was categorised into two sub-groups according to the Joint Scientific Statement of MetS [17]. To avoid the overlap of “high-normal HDL” and “normal HDL” in the Joint Scientific Statement criteria, we defined high-normal HDL as HDL ≥ 60 mg/dL; normal HDL as 40 mg/dL ≤ HDL < 60 mg/dL for men and 50 mg/dL ≤ HDL < 60 mg/dL for women; low HDL as HDL < 40 mg/dL for men and HDL < 40 mg/dL for women.

### Statistical analysis

Categorical data are presented as percentages, and continuous data as mean plus standard deviation (SD). The five components’ statuses were analysed at baseline and follow-up. We performed ARM to analyse the changes in each MetS component or combinations thereof during the five-year period of this cohort study. As the subjects at baseline were people with two or fewer MetS components, there were 16 (*i.e*. ${C}_5^0+{C}_5^1+{C}_5^2$) possible statuses at baseline, with 32 (*i.e*. 2^5^) possible statuses at follow-up. Theoretically there were 512 (*i.e*. 16 × 32) possible transitions between statuses from baseline to follow-up. The data are presented as transitions of statuses that changed from one risk factor to another, with “healthy” defined as being that state free of MetS components.

A few values from biochemical sera were missing, thus a multiple imputation (MI) was performed to impute the missing information. According to the data distribution, the Markov chain Monte Carlo (MCMC) method was chosen to avoid the loss of generality. All analyses were conducted using SAS software package (Version 9.2; SAS Institute, Chicago, IL, USA). A 2-sided *α* of 0.05 was considered statistically significant. Bonferroni adjustment of critical p-values was adopted when performing multiple comparisons.

### Ethics statement

This study was approved by the Ethics Committee of the Capital Medical University of China, Beijing, and performed in accordance with the principles of the Declaration of Helsinki (Reference No. 2013SY26).

### Consent

Written informed consent was obtained from each patient for publication of this report and any accompanying images.

## Results

At baseline, of the 4,905 subjects, 45.3% were men with a median age of 40.0 years (41.2 ± 11.0), while 54.7% were women with a median age of 42 years (41.7 ± 10.2). The follow-up interval was 4.78 ± 0.41 years for men and 4.81 ± 0.39 years for women, respectively. The baseline characteristics are summarised in Table 1.

**Table 1:** **The baseline characteristics of 20 to 65 years old subjects (n = 4 905)**

**Variables**	**Total**	**Men**	**Women**	***t*** **value**	***P*** **value**
N	4905	2222	2683	-	-
Age (years)	41.5 ± 10.6	41.2 ± 11.0	41.7 ± 10.2	1.41	0.1582^a^
HDL (mmol/L)	22.6 ± 2.6	1.4 ± 0.3	1.6 ± 0.3	36.04	<0.0001^a^
SBP (mmHg)	108.2 ± 10.2	111.1 ± 9.0	105.7 ± 10.4	-19.44	<0.0001^a^
DBP (mmHg)	71.4 ± 7.2	73.5 ± 6.4	69.7 ± 7.4	-19.10	<0.0001^a^
TG (mmol/L)	4.9 ± 0.4	1.0 ± 0.3	0.8 ± 0.3	-20.62	<0.0001^a^
FPG (mmol/L)	0.9 ± 0.3	4.9 ± 0.4	4.9 ± 0.4	-2.57	0.0103^a^
BMI (kg/m2)	1.5 ± 0.3	23.5 ± 2.5	21.8 ± 2.5	-22.95	<0.0001^a^
Elevated HDL (%)	281(5.73)	172(7.74)	109 (4.06)	30.4467	<0.0001^b^
Elevated BP (%)	42(0.86)	24(1.08)	18(0.67)	2.3975	0.1215^b^
Elevated TG (%)	20(0.41)	13(0.59)	7(0.26)	3.1450	0.0762^b^
Elevated FPG (%)	19(0.39)	10(0.45)	9(0.34)	0.4137	0.5201^b^
Elevated BMI (%)	17(0.35)	11(0.50)	6(0.22)	2.5924	0.1074^b^

*MetS* metabolic syndrome, *BMI* body mass index, *SBP* systolic blood pressure, *DBP* diastolic blood pressure, *FPG* fasting plasma glucose, *TG* triglycerides.

^a^Determined by two-sample t test.

^b^Determined by *χ* ^2^ test.

**Figure 1:**
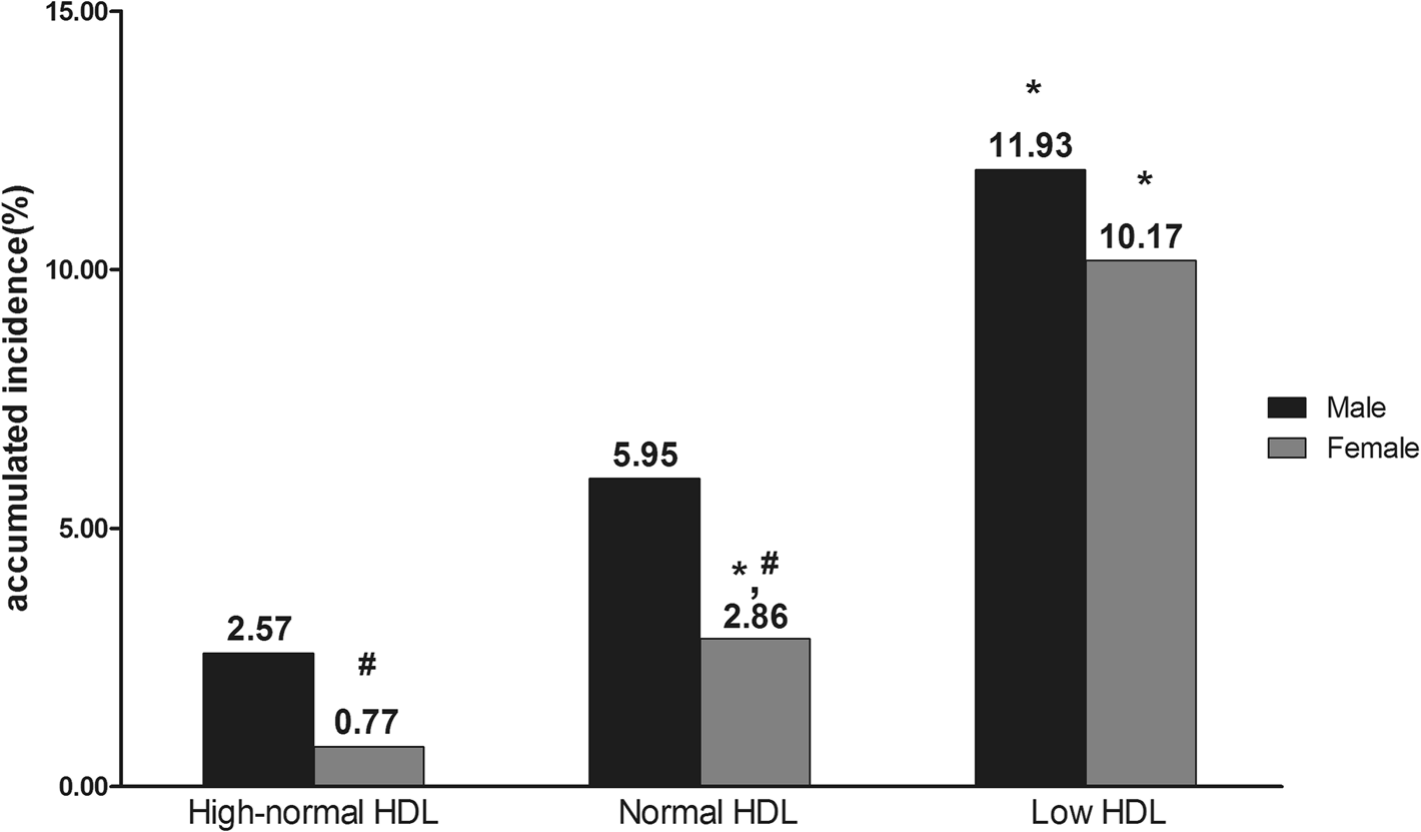
The accumulated incidence of MetS in subjects aged 20-44, stratified by gender and high-density lipoprotein level. *Compared with high-normal HDL group of same gender using χ^2^ test, P < 0.025. ^#^Compared with male counterpart using χ^2^ test, P < 0.05.

**Figure 2:**
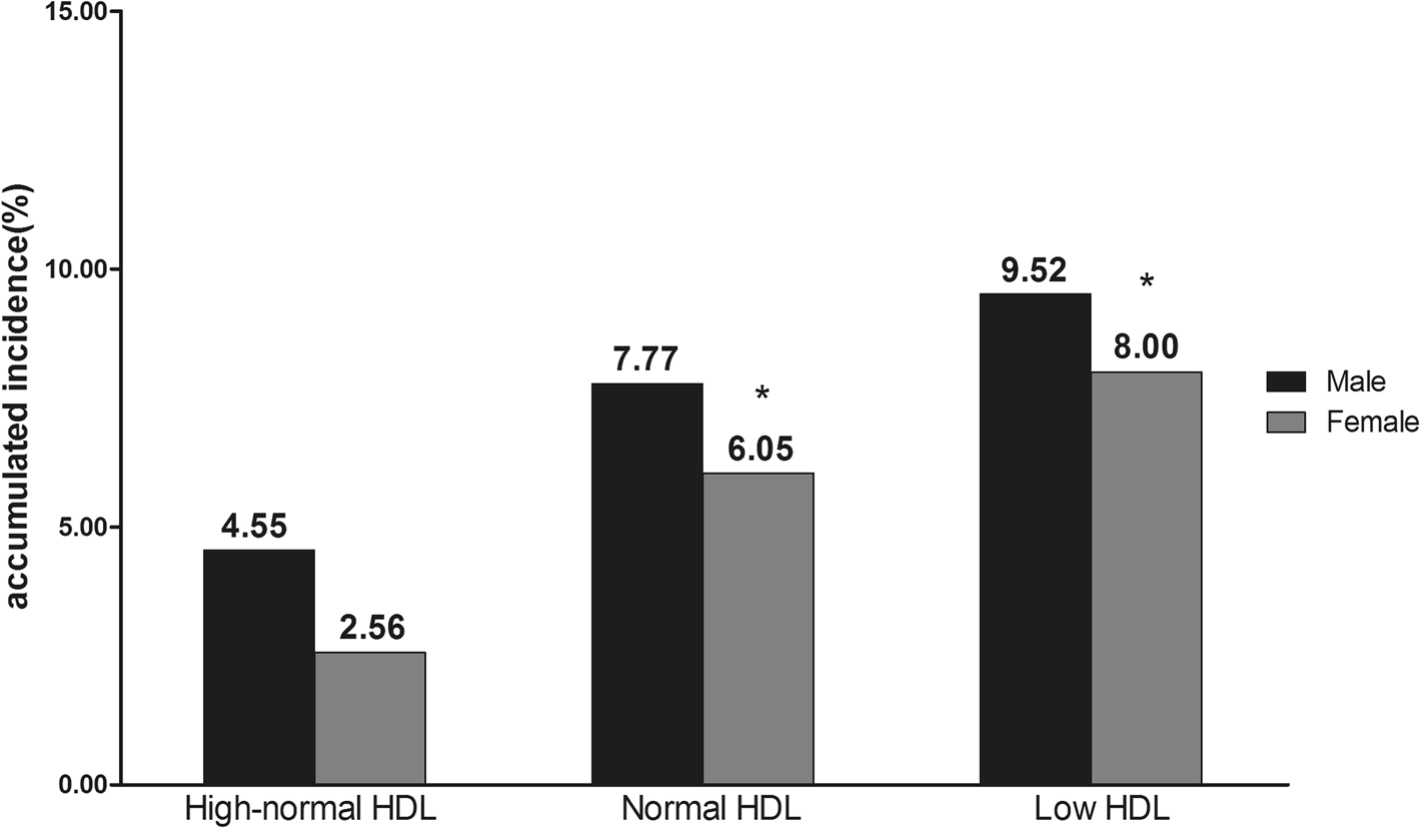
The accumulated incidence of MetS in subjects aged 45-65, stratified by gender and high-density lipoprotein level. *Compared with high-normal HDL group of same gender using χ^2^ test, P < 0.025.

The components of MetS in the population at baseline and follow-up were grouped by HDL level at baseline (Tables 2, 3, 4 and 5). Subjects were divided into two age groups (20 to 44 (young) and 45 to 65 (old)). The incidence of MetS at follow-up was analysed by gender and HDL level at baseline (Figures 1 and 2). Young men with high-normal and normal HDL levels had a higher incidence of MetS compared with women in the same groups (2.57% *versus* 0.77%, and 5.95% *versus* 3.45%, respectively). The numbers of young men and young women were almost the same in the low HDL group (11.93% *versus* 10.17%). Whereas the numbers of old men and old women were almost the same in the high-normal, normal, and low HDL groups (4.55% *versus* 2.56%, 7.77% *versus* 6.05%, and 9.52% *versus* 8.00%, respectively).

**Table 2:** **The profile of MetS components in men aged 20 to 44 of different high density lipoprotein group at baseline (n = 1 356)**

**Variable**	**High-normal HDL (n = 272)**			**Normal HDL (n = 975)**			**Low HDL (n = 109)**		
	**Baseline**	**Follow-up**	**P-value**	**Baseline**	**Follow-up**	**P-value**	**Baseline**	**Follow-up**	**P-value**
HDL (mmol/L)	1.75 ± 0.20	1.46 ± 0.30	<0.0001	1.27 ± 0.13	1.22 ± 0.24	<0.0001	0.98 ± 0.08	1.12 ± 0.21	<0.0001
SBP (mmHg)	110.18 ± 9.38	116.44 ± 11.63	<0.0001	110.76 ± 8.81	117.82 ± 12.11	<0.0001	110.50 ± 8.09	121.48 ± 13.67	<0.0001
DBP (mmHg)	72.62 ± 6.48	71.65 ± 8.95	0.0824	72.91 ± 6.50	72.67 ± 9.07	0.4203	72.63 ± 6.05	74.37 ± 10.86	0.0836
TG (mmol/L)	0.89 ± 0.31	1.19 ± 0.77	<0.0001	1.00 ± 0.34	1.35 ± 0.76	<0.0001	1.12 ± 0.38	1.61 ± 0.86	<0.0001
FPG (mmol/L)	4.86 ± 0.47	5.01 ± 0.42	<0.0001	4.87 ± 0.40	5.10 ± 0.48	<0.0001	4.78 ± 0.49	5.16 ± 0.64	<0.0001
BMI (kg/m2)	22.19 ± 2.59	23.29 ± 2.93	<0.0001	23.44 ± 2.46	24.39 ± 2.66	<0.0001	24.07 ± 2.43	25.38 ± 2.65	<0.0001

*HDL* high density lipoprotein*, SBP* systolic blood pressure, *DBP* diastolic blood pressure, *TG* triglycerides, *FPG* fasting plasma glucose.

*BMI* body mass index. *P*-value is based on paired t-test.

**Table 3:** **The profile of MetS components in men aged 45 to 65 of different high density lipoprotein group at baseline (n = 866)**

**Variable**	**High-normal HDL (n = 198)**			**Normal HDL (n = 605)**			**Low HDL (n = 63)**		
	**Baseline**	**Follow-up**	**P-value**	**Baseline**	**Follow-up**	**P-value**	**Baseline**	**Follow-up**	**P-value**
HDL (mmol/L)	1.76 ± 0.22	1.51 ± 0.34	<0.0001	1.28 ± 0.14	1.27 ± 0.26	0.1199	0.98 ± 0.11	1.17 ± 0.31	<0.0001
SBP (mmHg)	111.99 ± 9.68	119.40 ± 13.96	<0.0001	111.90 ± 8.98	120.90 ± 15.08	<0.0001	111.92 ± 9.08	120.36 ± 13.02	<0.0001
DBP (mmHg)	74.14 ± 6.36	73.40 ± 9.74	0.3292	74.64 ± 6.21	75.01 ± 10.39	0.3860	74.04 ± 6.62	76.16 ± 9.92	0.1110
TG (mmol/L)	1.01 ± 0.34	1.27 ± 0.85	<0.0001	1.11 ± 0.30	1.32 ± 0.58	<0.0001	1.14 ± 0.37	1.35 ± 0.54	0.0020
FPG (mmol/L)	4.97 ± 0.38	5.21 ± 0.51	<0.0001	4.95 ± 0.39	5.26 ± 0.72	<0.0001	4.70 ± 0.52	5.21 ± 0.60	<0.0001
BMI (kg/m2)	23.18 ± 2.52	23.59 ± 2.67	0.0024	23.96 ± 2.27	24.33 ± 2.48	<0.0001	24.29 ± 2.17	24.69 ± 2.31	0.0810

*HDL* high density lipoprotein, *SBP* systolic blood pressure, *DBP* diastolic blood pressure, *TG* triglycerides, *FPG* fasting plasma glucose.

*BMI* body mass index. *P*-value is based on paired t-test.

**Table 4:** **The profile of MetS components group in women aged 20 to 44 of different high density lipoprotein at baseline (n = 1 595)**

**Variable**	**High-normal HDL (n = 907)**			**Normal HDL (n = 629)**			**Low HDL (n = 59)**		
	**Baseline**	**Follow-up**	**P-value**	**Baseline**	**Follow-up**	**P-value**	**Baseline**	**Follow-up**	**P-value**
HDL (mmol/L)	1.83 ± 0.23	1.65 ± 0.33	<0.0001	1.41 ± 0.08	1.40 ± 0.25	0.0576	1.13 ± 0.14	1.40 ± 0.33	<0.0001
SBP (mmHg)	108.65 ± 10.21	108.83 ± 11.96	<0.0001	108.30 ± 10.32	110.07 ± 13.05	<0.0001	110.03 ± 10.08	112.75 ± 14.99	0.0007
DBP (mmHg)	71.51 ± 7.10	67.65 ± 8.41	0.0460	71.47 ± 7.39	67.95 ± 9.51	0.0813	72.81 ± 7.39	70.97 ± 9.84	0.1093
TG (mmol/L)	0.91 ± 0.31	0.87 ± 0.41	<0.0001	1.00 ± 0.32	0.99 ± 0.48	<0.0001	0.90 ± 0.31	1.17 ± 1.03	0.0455
FPG (mmol/L)	4.89 ± 0.35	5.00 ± 0.45	<0.0001	4.88 ± 0.36	5.02 ± 0.42	<0.0001	4.79 ± 0.39	4.99 ± 0.41	0.0001
BMI (kg/m2)	22.47 ± 2.44	21.54 ± 2.68	<0.0001	23.02 ± 2.33	22.18 ± 2.83	<0.0001	23.53 ± 2.43	22.42 ± 2.81	0.0465

*HDL* high density lipoprotein, *SBP* systolic blood pressure, *DBP* diastolic blood pressure, *TG* triglycerides, *FPG* fasting plasma glucose.

*BMI* body mass index. *P*-value is based on paired t-test.

**Table 5:** **The profile of MetS components in women aged 45 to 65 of different high density lipoprotein group at baseline (n = 1 088)**

**Variable**	**High-normal HDL (n = 625)**			**Normal HDL (n = 413)**			**Low HDL (n = 50)**		
	**Baseline**	**Follow-up**	***P*** **-value**	**Baseline**	**Follow-up**	***P*** **-value**	**Baseline**	**Follow-up**	***P*** **-value**
HDL (mmol/L)	1.76 ± 0.22	1.69 ± 0.32	<0.0001	1.28 ± 0.14	1.46 ± 0.29	0.0003	0.98 ± 0.11	1.58 ± 0.27	<0.0001
SBP (mmHg)	111.88 ± 9.68	116.32 ± 15.01	<0.0001	111.90 ± 8.98	118.10 ± 15.19	<0.0001	111.92 ± 9.08	119.96 ± 14.08	<0.0001
DBP (mmHg)	74.14 ± 6.36	71.52 ± 9.77	0.9928	74.64 ± 6.21	72.79 ± 9.66	0.0064	74.04 ± 6.62	72.86 ± 10.70	0.9747
TG (mmol/L)	1.01 ± 0.34	1.14 ± 0.57	<0.0001	1.11 ± 0.30	1.21 ± 0.53	<0.0001	1.14 ± 0.37	1.24 ± 0.58	<0.0001
FPG (mmol/L)	4.97 ± 0.38	5.10 ± 0.46	<0.0001	4.95 ± 0.39	5.16 ± 0.50	<0.0001	4.70 ± 0.52	5.19 ± 0.49	<0.0001
BMI (kg/m2)	23.18 ± 2.52	22.95 ± 2.76	<0.0001	23.96 ± 2.27	23.34 ± 2.49	0.0008	24.29 ± 2.17	23.50 ± 2.79	0.9406

*HDL* high density lipoprotein, *SBP* systolic blood pressure, *DBP* diastolic blood pressure, *TG* triglycerides, *FPG* fasting plasma glucose.

*BMI* body mass index. *P*-value is based on paired t-test.

**Figure 3:**
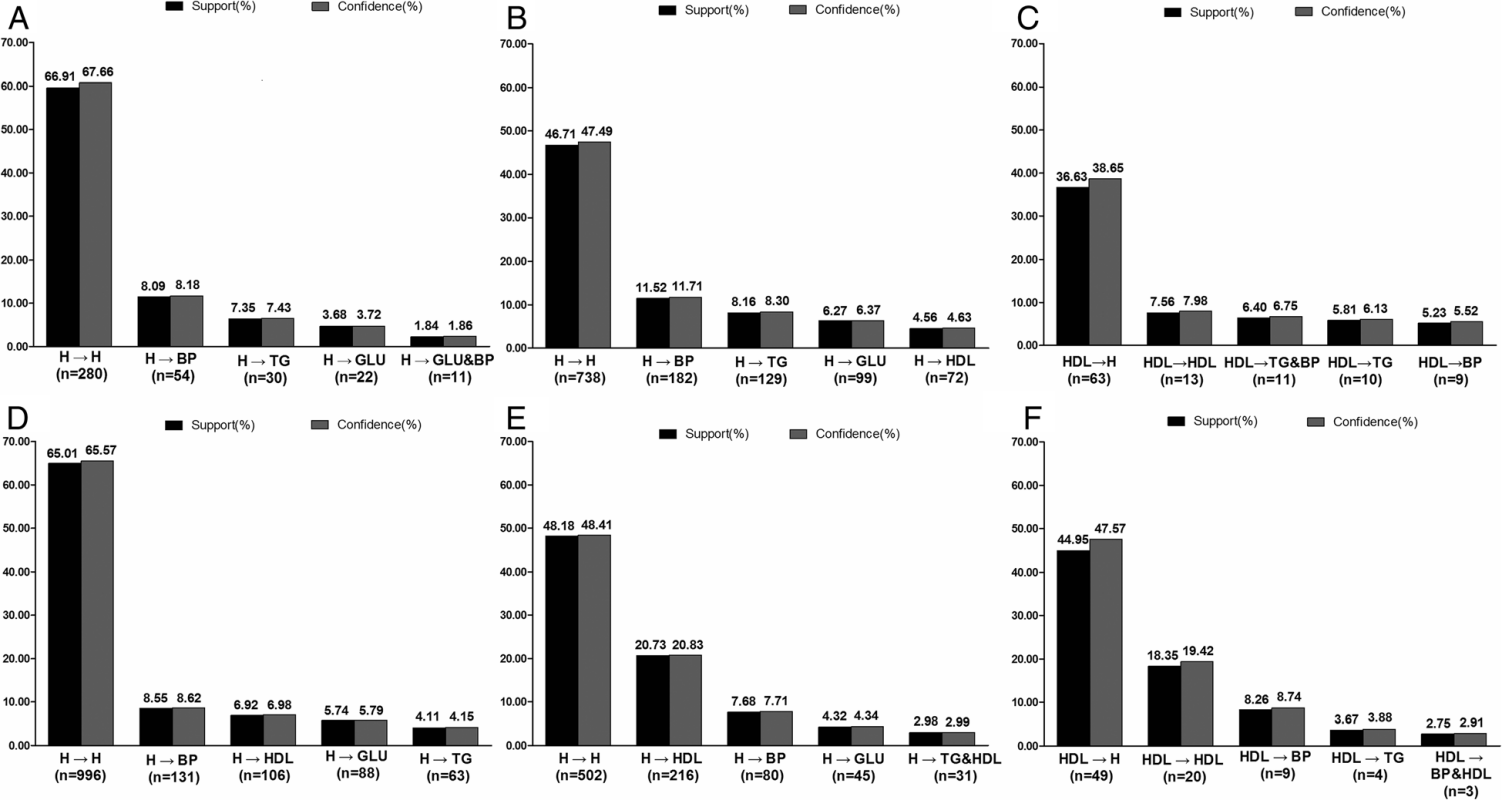
The support and confidence rate of the top five transitions in all subjects from 2007/2008 to 2011/2012 stratified by sex and high-density lipoprotein groups. **(A)** in high-normal HDL group of males; **(B)** in normal HDL group of males; **(C)** in low HDL group of males; **(D)** in high-normal HDL group of females; **(E)** in normal HDL group of females; **(F)** in low HDL group of females. Abbreviations: H, health, with the absence of any MetS components; BP, high blood pressure; TG, raised triglycerides level; GLU, high fasting plasma glucose; HDL, increased high-density lipoprotein level.

The five most common transitions in each sub-group were identified (Figure 3). As the sample size was too small after grouping by age, the association rule was applied without sub-stratification of age. The support rate of transitions (defined as the percentage changing from initial status to another status via all possible transitions) was examined in the period 2007/2008 to 2011/2012 in the different sub-groups at baseline. For visual simplicity, the five most commonly observed transitions are shown in the relevant parts of Figure 3. The most common transition in both genders with normal-high and normal HDL was “healthy” to “healthy”, whereas for the low HDL group, the main transition was from “low HDL” to “healthy”. The rates of transition are shown for each gender and HDL group. The confidence rate represents how many cases transitioned within a certain status. For example, 470 males were initially healthy at baseline, and 280 (60.74%) of these stayed healthy, while 54 (11.71%) transitioned to hypertension at the end of the observation period.

## Discussion

This cohort study, comprising 4,905 subjects in Beijing Tongren and Beijing Xiaotangshan Hospitals, focused on investigating how the incidence of MetS changed relative to HDL level and which MetS components tend to emerge and change during the 5-year follow-up period. Our study identified that the lower the HDL at baseline, the higher the incidence of MetS at follow-up. We found that women with different levels of HDL would develop different combinations of MetS components. On analysing the change in MetS components, we identified that those women with low HDL tended to have elevated blood pressure as the most common additional MetS component, while those women with higher HDL tended to have elevated HDL as the most common new-onset MetS component. Men tended to have elevated blood pressure as the most common additional and new-onset MetS component.

The HDL of people with normal-high and normal high-density lipoprotein at baseline tended to decrease with time, while the HDL increased in those subjects with low high-density lipoprotein, according to Tables 2, 3, 4 and 5. Subjects with low high-density lipoprotein may be alert to their health as health education and promotion programmes are available through various media in Beijing, possible actions on exercise and diet could be intentionally or unintentionally taken, therefore their HDL is controlled or even higher.

An association between low HDL and MetS has been reported as the most prominent new onset MetS component, or even serves as a key component of predicting cardiovascular and diabetes risk [9,13,23]. Several studies with structural equation modelling showed that low HDL might play both a direct and indirect role in the progression of MetS [24-26]. However, physiologically, it is not easy to connect low HDL with MetS. In the present study, we found that people with high-normal and normal HDL tended to have a relatively lower incidence of MetS after five years when compared with people with low HDL, and were less susceptible to developing the disorder. Most subjects who started off as healthy remained healthy, similar to a previous study amongst a German population [13]. However, lowered HDL tended to be the first risk factor of MetS for people with normal HDL, which is roughly confirmed in another population [13]. About 42% of the subjects with low HDL returned to “healthy”, while 12% continued to remain in the same condition. Low HDL-C level has been found to be independently and significantly related to myocardial infarction or stroke in patients with MetS, thus multi-factor treatment strategies, including strict life style change, should be made to improve dyslipidemia in MetS and decrease the residual risk for CVD in MetS [5,27].

The components of MetS tended to cluster in a way which varied from one population to another [13,28]. In our study, people with low HDL tended to have raised blood pressure as a secondary risk factor. This is roughly consistent with other studies, which found that elevated blood pressure was the second most important component of MetS and people with MetS tended to have hypertension [14,23]. Low HDL seemed to be related to each of the other four components. It is mainly a consequence of systemic low-grade inflammation and apo A-I dysfunction. In addition to the five components of MetS, pro-thrombotic and pro-inflammatory states are essential features based on the evidence of impaired function of HDL and apo A-I particles are discernible by biological evidence of functional defectiveness via outcomes studies and/or correlations with inflammatory and anti-inflammatory biomarkers [29]. The aggregation to lipoprotein (Lp) (a) of apo A-I underlies HDL dysfunction, and is an independent risk factor of magnitude similar to conventional components of MetS [30]. Several studies showed that the steep increase in dyslipidemia could be the reason for the growing prevalence of diabetes and *vice versa* [23,31]. Dyslipidemia in patients with MetS may be caused by a combination of increased catabolism of HDL-apo A-I particles, overproduction of very LDL apo B, and decreased catabolism of apo B containing particles: these abnormalities may be consequences of insulin resistance [32]. An important link between obesity, the metabolic syndrome, and dyslipidemia, seems to be the development of insulin resistance in peripheral tissues leading to an enhanced hepatic flux of fatty acids from dietary sources, intravascular lipolysis, and from adipose tissue resistant to the antilipolytic effects of insulin [32]. Previous reports indicated that pro-inflammatory state and oxidative stress are crucial for evaluating cardiometabolic risk. Factors such as creatinine, platelet-activating factor acetyl hydrolase, thyroid stimulating hormone, acetylation-stimulating protein, asymmetric dimethylarginine, and serum lipoprotein (Lp) (a) are key to triggering systemic low-grade inflammation and enhanced autoimmune reactions, which may induce low HDL and metabolic syndrome [33].

In most circumstances, “healthy” was the predominant status, and subjects with a single MetS component tended to return to a “healthy” status. Low HDL seemed to be a crucial status for MetS prevention. Since dyslipidemia has low rates of awareness, treatment, and control among Chinese adults, it is an important preventable risk factor for MetS and CVD events [34].

The strengths of this study were that it was a longitudinal study over five years in a Chinese population with data subject to relatively good quality control. There were some limitations to this study. The first limitation of this study is the relatively small sample that might not be sufficiently representative of the general adult population and the demographics and referral source may limit the generalisation of the results. Further studies using the general population would be desirable. Secondly, information about lifestyles was not available, but lifestyle variables will be included in further studies. The third limitation is the lack of WC measurements as an indicator of central obesity. However, BMI > 30 kg/m2 was used as a substitute for obesity [19]. Several studies have indicated that two measures of BMI and WC are closely correlated [18,35]. Most individuals with an abnormal BMI also have an abnormal WC.

## Conclusions

The incidence of MetS increases with the reduction of HDL over time. People with high-normal and normal HDL were less susceptible than people with higher HDL to developing MetS. Although most cases in the normal HDL group stayed “healthy”, they tended to have decreased HDL as their initial sign of MetS. Low HDL was a crucial status for MetS prevention as some of the cases were able to return to “healthy”. In the low HDL group, elevated blood pressure was a secondary risk factor. More effective risk mitigation strategies are needed for people with low HDL to prevent those developing MetS.

## Abbreviations

HDL: High-density lipoprotein
MetS: Metabolic syndrome
CVD: Cardiovascular disease
DM: Diabetes mellitus
ARM: Association rule mining
BP: Blood pressure
SBP: Systolic blood pressure
DBP: Diastolic blood pressure
BMI: Body mass index
WC: Waist circumference
MBA: Market basket analysis
SD: Standard deviation
MCMC: Markov chain Monte Carlo
